# Long-term air pollution and adverse meteorological factors might elevate the osteoporosis risk among adult Chinese

**DOI:** 10.3389/fpubh.2024.1361911

**Published:** 2024-01-29

**Authors:** Hong Sun, Yanan Wan, Xiaoqun Pan, Wanxi You, Jianxin Shen, Junhua Lu, Gangfeng Zheng, Xinlin Li, Xiaoxi Xing, Yongqing Zhang

**Affiliations:** ^1^Jiangsu Provincial Center for Disease Control and Prevention, Nanjing, Jiangsu, China; ^2^Luhe District Center for Disease Control and Prevention, Nanjing, Jiangsu, China; ^3^Wujiang District Center for Disease Control and Prevention, Suzhou, Jiangsu, China; ^4^Chongchuan District Center for Disease Control and Prevention, Nantong, Jiangsu, China; ^5^Jingjiang Center for Disease Control and Prevention, Taizhou, Jiangsu, China; ^6^Nantong Center for Disease Control and Prevention, Nantong, Jiangsu, China; ^7^Quanshan District Center for Disease Control and Prevention, Xuzhou, Jiangsu, China

**Keywords:** bone mineral density, osteoporosis prevalence, particulate matter, lag times, susceptibility

## Abstract

**Objective:**

This study aims to investigate the relationship between exposure to air pollution and adverse meteorological factors, and the risk of osteoporosis.

**Methods:**

We diagnosed osteoporosis by assessing bone mineral density through Dual-Energy X-ray absorptiometry in 2,361 participants from Jiangsu, China. Additionally, we conducted physical examinations, blood tests, and questionnaires. We evaluated pollution exposure levels using grid data, considering various lag periods (ranging from one to five years) based on participants’ addresses. We utilized logistic regression analysis, adjusted for temperature, humidity, and individual factors, to examine the connections between osteoporosis and seven air pollutants: PM₁, PM₂.₅, PM₁₀, SO₂, NO₂, CO, and O₃. We assessed the robustness of our study through two-pollutant models and distributed lag non-linear models (DLNM) and explored susceptibility using stratified analyses.

**Results:**

In Jiangsu, China, the prevalence of osteoporosis among individuals aged 40 and above was found to be 15.1%. A consistent association was observed between osteoporosis and the five-year average exposure to most pollutants, including PM₂.₅, PM₁₀, CO, and O₃. The effects of PM₁₀ and CO remained stable even after adjusting for the presence of a second pollutant. However, the levels of PM₁ and PM₂.₅ were significantly influenced by O₃ levels. Individuals aged 60 and above, those with a BMI of 25 or higher, and males were found to be more susceptible to the effects of air pollution. Interestingly, males showed a significantly higher susceptibility to PM₁ and PM₂.₅ compared to females. This study provides valuable insights into the long-term effects of air pollution on osteoporosis risk among the adult population in China.

**Conclusion:**

This study indicates a potential association between air pollutants and osteoporosis, particularly with long-term exposure. The risk of osteoporosis induced by air pollution is found to be higher in individuals aged 60 and above, those with a BMI greater than 25, and males. These findings underscore the need for further research and public health interventions to mitigate the impact of air pollution on bone health.

## 1 Introduction

Osteoporosis is a systemic skeletal disorder characterized by reduced bone mass and microscopic structural deterioration of bone tissue, leading to increased fragility of bones and a higher risk of fractures (1). In older adult individuals, particularly among women, bone pain and fractures are common symptoms of osteoporosis, which can potentially lead to disability or even death. This disease imposes a significant burden on healthcare systems, and with the increasing old population, this burden continues to grow (2). Taking China as an example, there were 411,000 cases of hip fractures in 2015, and it is projected to increase to one million by 2050 (3). Based on an osteoporosis epidemiological study conducted in China, which surveyed 20,416 individuals, the prevalence of osteoporosis among adults aged 40 and above was 5.0% for men and 20.6% for women (4). When combining this data with the sixth Chinese national census (55,191,915 men aged 40 and above and 53,935,201 women aged 40 and above), we estimated that there are 13.87 million osteoporosis patients among the Chinese population aged 40 and above. Therefore, the implementation of comprehensive early prevention and treatment measures for osteoporosis has become extremely urgent and necessary.

Air pollution is recognized a global health challenge (5). As early as 1985, researchers suggested a potential link between air pollution and osteoporosis (6). The Oslo Health Study (7) initially identified a weak but significant negative correlation between a 10-year average of air pollution indicators and whole-body bone density. Recent evidence from the analysis of 9.2 million U.S. health insurance records (8) and data from over 40,000 individuals in South Korea’s health insurance database (9) indicates a close association between increased PM_2.5_ concentrations and higher rates of hospitalization due to fractures in the older adults, suggesting a connection between air pollution and osteoporosis. An analysis of data from 341,000 participants in the UK Biobank also suggests that exposure to higher levels of air pollution is associated with lower bone mineral density and an increased risk of osteoporosis (10). However, despite over four decades of research, the existing evidence regarding the relationship between outdoor air pollution exposure and osteoporosis-related outcomes remains scattered and inconclusive (11). Meta-analyses of limited studies indicate heterogeneous results regarding the association between air pollution exposure and osteoporosis (11), and the observed inconsistencies between studies may be attributed to heterogeneity in participant characteristics, study designs, and statistical issues (12). Furthermore, recent studies have adopted diverse lag periods for long-term exposure, while a considerable number have omitted adjustments for meteorological variables, which may constitute significant contributors to the disparate research findings.

Therefore, we conducted a retrospective cohort study in Jiangsu, China, and assessed the 5-year daily exposure of the survey participants to air pollutants and meteorological factors. Our study aimed to assess the impact of various air pollutants and adverse meteorological factors, including three kinds of Particulate Matter with different aerodynamic diameters (PM₁, PM₂.₅, PM₁₀), Nitrogen Dioxide (NO₂), Sulfur Dioxide (SO₂), Carbon Monoxide (CO), and ozone (O₃), as well as high humidity and solar irradiation, on the risk of osteoporosis. This research is crucial in understanding the environmental factors contributing to osteoporosis and informing public health interventions.

## 2 Materials and methods

### 2.1 Study population

The study population for this cohort research constitutes a subset of the China National Epidemiological Survey on Osteoporosis, conducted in 2017 (4). This national study aimed to investigate the prevalence of osteoporosis and its associated risk factors. Our study was conducted in Jiangsu Province, located in the eastern part of China, from March to July 2018. Jiangsu Province is characterized by predominantly flat terrain and is considered an economically developed region in China. The survey encompassed six cities within Jiangsu Province, each representing various urban environments (Supplementary Figure S1). We employed a multi-stage, stratified cluster random sampling approach for our sampling method. In each surveyed area, we used a Probability Proportional to Size (PPS) sampling method to randomly select four townships or streets, each providing two administrative villages or communities. Afterward, we randomly selected one resident group from each administrative village or community, with each group comprising a minimum of 50 participants aged 40 years and older who met the eligibility criteria on bone mineral density measurements. Exclusion criteria included individuals diagnosed with metabolic bone diseases such as hyperthyroidism, hyperparathyroidism, renal failure, malabsorption syndrome, alcoholism, chronic colitis, multiple myeloma, leukemia, or chronic arthritis, as well as pregnant individuals.

### 2.2 Osteoporosis assessment

We conducted bone mineral density (BMD) measurements, including lumbar spine (L1 to L4), femoral neck, and total hip, using Hologic scanners (Hologic Inc) or GE-Lunar scanners (GE Healthcare) via dual-energy X-ray absorptiometry (DXA). Quality control procedures were rigorously implemented, encompassing the scanning of a standardized European Spine Phantom (ESP) ten times to calibrate each DXA scanner utilized during participant examinations. This meticulous calibration process was pivotal in guaranteeing the uniformity and accuracy of Bone Mineral Density (BMD) measurements, an essential factor in the scoring and analysis for this study. It underscored our commitment to maintaining consistency in data collection and analysis, thereby fortifying the reliability of our findings. Osteoporosis diagnosis adhered to the criteria set by the World Health Organization, calculated as T-score = (BMD – gender-specific peak BMD) / (SD of gender-specific peak BMD). Individuals with T-scores of −2.5 or lower at any site (L1 to L4, femoral neck, or total hip) were classified as having osteoporosis (13). The data calculation methods in this study align with those utilized in the previous study (4).

### 2.3 Exposure assessment

Daily ambient air pollution data, which included PM₁, PM₂.₅, PM₁₀, SO₂, NO₂, CO, and O₃, were obtained from the ChinaHighAirPollutants dataset, accessible at https://weijing-rs.github.io/product.html. This dataset was generated through a combination of artificial intelligence models, ground measurements, satellite remote sensing products, and atmospheric reanalysis. It offered comprehensive spatiotemporal coverage across China during the study period, with a spatial resolution of 1 × 1 km for PM and O₃, and 10 × 10 km for SO₂, NO₂, and CO. The reliability of the exposure assessment has been validated in our previous studies (14, 15).

We collected daily pollution and meteorological exposure data for participants’ residential locations from 2013 to 2018. Based on each participant’s survey date, we computed annual average exposure levels for the year preceding the survey (lag0) up to 5 years before the survey (lag4). Additionally, we calculated exposure averages from 2 years before the survey (lag01) to 5 years before the survey (lag04). Note that data for PM₁ in 2013 were missing, resulting in a one-year shorter exposure period, with a maximum of 4 years.

### 2.4 Covariates

Meteorological data, which included air temperature (°C) and relative humidity (%), were sourced from the China Meteorological Administration Land Data Assimilation System (CLDAS version 2.0) at a spatial resolution of 0.0625° × 0.0625° (16, 17). Additionally, we retrieved data on Erythemal Daily Dose (EDD) from the Dutch-Finnish Ozone Monitoring Instrument (OMI) Level 2 UV irradiance products (OMUVB V003) at a resolution of 13 km × 24 km (18). EDD represents the cumulative UV radiation exposure individuals receive in a day, with the potential to cause skin erythema (sunburn) (19). It is measured in J/m^2^ and is commonly used to assess the risk of skin damage due to UV radiation. The OMI spectrometer, hosted by the NASA Aura satellite, observes nadir views and records ultraviolet wavelengths ranging from 270 to 380 nm. We calculated daily mean EDD levels for specific locations by averaging EDD values from corresponding OMI pixels within those areas. The methodology used for assessing exposure to meteorological factors aligned with the approach employed for air pollutants. Individual covariates, such as gender, age, and body mass index (BMI), were collected through questionnaires and physical examinations.

### 2.5 Statistical analysis

We conducted Spearman’s correlation tests to explore the relationships between air pollutant exposures and meteorological factors. Subsequently, logistic regression models were employed to assess the exposure-response associations for PM₁, PM₂.₅, PM₁₀, SO₂, NO₂, CO, and O₃ exposures concerning osteoporosis incidents. Using a stepwise selection approach, individual factors such as BMI (body mass index), age, and gender were incorporated into the model. Unit-Based Root Expected Logarithmic Prediction (UBRE) is used to assess the goodness of fit of a model. These logistic regression models allowed us to estimate the percentage changes in the odds of osteoporosis incidents, expressed as ([odds ratio – 1] * 100%), across various exposure levels. Alongside these estimates, we calculated corresponding 95% confidence intervals (CIs) and determined the percentage change in the odds of osteoporosis for a unit increase in exposure. In constructing these models, we utilized natural cubic spline functions (with 3 degrees of freedom [df]) to portray the exposure to each specific pollutant, thus forming exposure-response curves. To ensure robustness, all models were adjusted for annual air temperature and relative humidity (RH), which were included as natural cubic spline functions (df = 3). Additionally, in two-pollutant models and stratified analyses, adjustments were made for additional variables, including EDD.

Furthermore, we performed a comprehensive stratified analysis based on age (<60, ≥60 years), gender (male, female), and BMI (<25, ≥25). Effect modifications were rigorously examined using two-sample z-tests, leveraging the stratification-specific point estimates (*β* = ln odds ratio) and their corresponding standard errors (SEs) (20):

$$z=\frac{\beta_{1}-\beta_{2}}{\sqrt{SE_{1}^{2}+SE_{2}^{2}}}$$

To ensure robustness, we conducted sensitivity analyses, including two-pollutant models for each of the seven air pollutants. These models integrated an additional set of pollutants for assessment, and we specifically utilized the likelihood ratio test to compare nested single-pollutant and two-pollutant models, aiming to discern differences between the models. We also considered the potential non-linear lag effects of pollutant exposure over different years. To do so, we used the Distributed Lag Non-Linear Model (DLNM) approach to assess the associations between osteoporosis occurrence and the seven pollutants, along with EDD, over various lag years.

All data analyses were performed using R version 4.3.1, with two-sided *p*-values, and statistical significance was set at *p* < 0.05.

## 3 Results

### 3.1 Study population and characteristics

A total of 2,399 individuals aged 40 and above participated in comprehensive health assessments and completed questionnaires, with 38 participants being excluded due to incomplete X-ray examinations. As shown in Table 1, a total of 2,361 individuals were included in this study, among whom 356 were diagnosed with osteoporosis, accounting for 15.1% of the total. A slightly higher proportion of participants were female, accounting for 57.8% of the sample. Nevertheless, the prevalence of osteoporosis among females was considerably higher, reaching 23.4%, which was 6.5 times greater than that among males (23.4/3.6). The mean age of the participants was 57.9 ± 9.7 years, with the osteoporosis group being older than the control group. Approximately 46.2% of the participants were aged 60 and above, with an osteoporosis prevalence of 24.7%, significantly higher than the prevalence in the age < 60 group (6.9%, 3.6 times higher). Regarding Body Mass Index (BMI), the participants had an average of 25.1 ± 3.4, with the BMI in the osteoporosis group being significantly lower than that in the control group. Among the surveyed individuals, 52.2% had a BMI below 25, and this group exhibited an osteoporosis prevalence of 19.2%, significantly higher than the prevalence among individuals with a BMI of 25 or greater (10.5%).

**Table 1:** Characteristics of the study population.

Characteristic	Total	Osteoporosis		*p*
		Yes	No	
Number	2,361	356 (15.1%)	2005 (84.9%)	
Gender				<0.01^a^
Male	996 (42.2%)	36 (3.6%)	960 (96.4%)	
Female	1,365 (57.8%)	320 (23.4%)	1,045 (76.6%)	
Age	57.9 ± 9.7	64.4 ± 7.8	56.7 ± 9.6	<0.01^a^
<60	1,270 (53.8%)	87 (6.9%)	1,183 (93.1%)	
≥60	1,091 (46.2%)	269 (24.7%)	822 (75.3%)	
BMI	25.1 ± 3.4	24.0 ± 3.6	25.3 ± 3.4	<0.01^a^
<25	1,232 (52.2%)	237 (19.2%)	995 (80.8%)	
≥25	1,129 (47.8%)	119 (10.5%)	1,010 (89.5%)	

Values are *n*, *n* (%) or means ± SD. ^a^The comparison is being made regarding the distribution differences of cases and non-cases across different gender, age, or BMI groups.

### 3.2 Exposure to air pollution and meteorological factors

**Figure 1:**
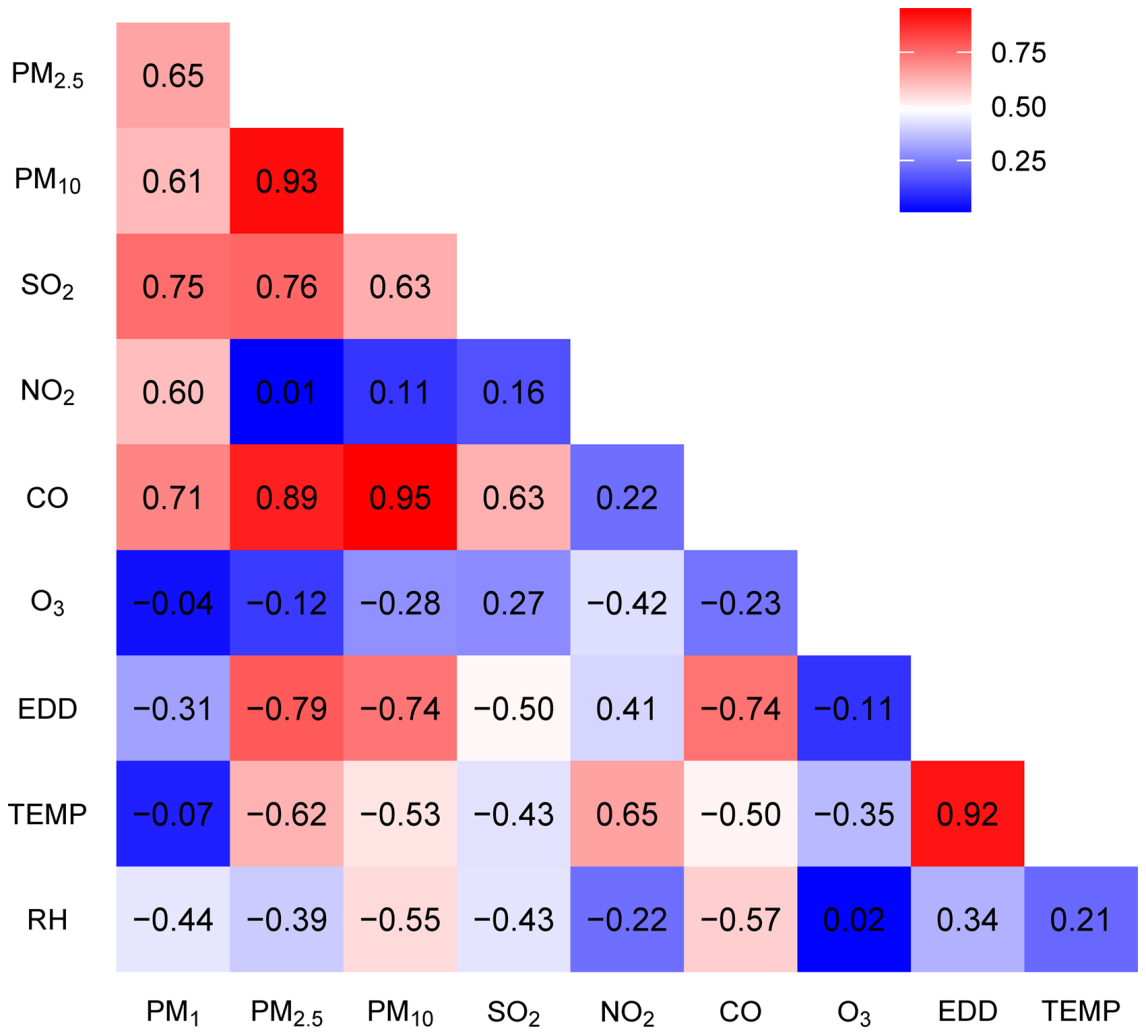
Correlation coefficients between seven air pollutants, solar radiation (EDD), temperature, and humidity.

In Table 2, we compiled data on the exposure of study participants to seven air pollutants (PM₁, PM₂.₅, PM₁₀, SO₂, NO₂, CO, O₃) and meteorological factors (temperature in °C, humidity in %, and Erythemal Daily Dose – EDD in J/m^2^) for various lag periods: the year before the survey (lag0), the average over the 2 years before the survey (lag01), and the average over the 5 years before the survey (lag04). Our findings indicate that between 2013 and 2018, the average concentrations of particulate matter in the surveyed areas gradually decreased. For instance, PM₁₀ decreased from 99.7 μg/m^3^ at lag04 (2013–2018) to 88.6 μg/m^3^ at lag0 (2017–2018). Similarly, the concentration of SO₂ during this period decreased from 25.7 to 16.5 μg/m^3^. In contrast, O₃ levels increased from 102.1 to 107.9 μg/m^3^. NO₂, CO, and meteorological factors remained relatively stable. Figure 1 illustrates the correlations among these factors in lag04 exposure, with PM₁₀, PM₂.₅, and CO exhibiting correlation coefficients exceeding 90%.

**Table 2:** Distribution of exposure to ambient air pollutants and meteorological conditions of study.

	^a^Lag0 year	^a^Lag01 year	Lag02 year	Lag03year	Lag04 year
	Mean (Range)	Mean (Range)	Mean (Range)	Mean (Range)	Mean (Range)
PM_1_ (μg/m^3^)	33.0 (26.8 to 43.2)	33.4 (28.8 to 41.3)	34.9 (30.5 to 41.6)	36.4 (32.5 to 41.5)	37.3 (32.4 to 43.2)
PM_2.5_ (μg/m^3^)	51.6 (39.9 to 70.3)	51.4 (40.6 to 66.6)	54.4 (45.3 to 66.7)	57.0 (48.4 to 67.9)	60.6 (52.2 to 71.6)
PM_10_ (μg/m^3^)	88.6 (66.3 to 118.4)	86.9 (65.8 to 116.1)	90.8 (71.6 to 118.9)	94.8 (77.1 to 121.7)	99.7 (83.4 to 125.3)
SO_2_ (μg/m^3^)	16.5 (13.1 to 20.8)	18.7 (14.0 to 25.7)	21.2 (15.8 to 30.6)	23.3 (17.2 to 34.3)	25.7 (18.7 to 38.4)
NO_2_ (μg/m^3^)	41.4 (36.8 to 48.7)	40.4 (35.1 to 46.9)	40.1 (33.1 to 46.2)	40.1 (31.7 to 46.2)	40.5 (32.1 to 46.0)
CO (mg/m^3^)	0.9 (0.7 to 0.9)	0.9 (0.7 to 1.0)	0.9 (0.7 to 1.1)	1.0 (0.7 to 1.2)	1.0 (0.7 to 1.2)
O_3_ (μg/m^3^)	107.9 (100.2 to 119.8)	106.0 (97.5 to 118.8)	104.4 (96.4 to 117.5)	103.2 (95.5 to 116.0)	102.1 (94.7 to 115.0)
Erythemal Daily Dose^b^ (J/m^2^)	2,395 (2,181 to 2,593)	2,333 (2,138 to 2,496)	2,318 (2,131 to 2,469)	2,294 (2,117 to 2,449)	2,322 (2,139 to 2,505)
Temperature (°C)	16.9 (15.7 to 18.2)	16.9 (15.8 to 18.2)	16.7 (15.7 to 17.9)	16.6 (15.6 to 17.8)	16.6 (15.7 to 17.8)
Humidity (%)	72.9 (66.9 to 75.8)	74.2 (69.1 to 77.2)	73.9 (68.4 to 76.9)	73.5 (68.0 to 76.3)	72.8 (67.7 to 75.5)

^a^Lag0 year: The average exposure in the year immediately before the survey day; Lag01 (~04) year: The average exposure over the 2 years (~5 years) preceding the survey day. ^b^Erythemal Daily Dose (J/m^2^): This refers to the total amount of ultraviolet (UV) radiation exposure that the Earth’s surface receives in a day, which may cause erythema (skin redness or sunburn). It represents the cumulative UV radiation dose within a day.

### 3.3 Lag and cumulative effects of pollutants on osteoporosis

**Figure 2:**
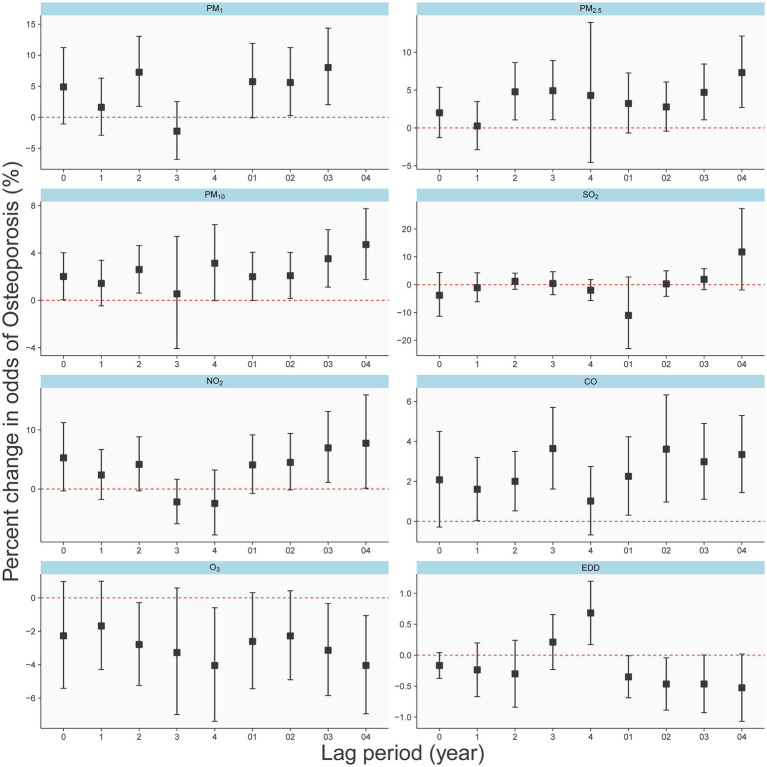
Different effects of air pollutants and EDD on the osteoporosis in single-year lag model and average lag model.

We identified humidity as a significant risk factor for osteoporosis (Supplementary Figure S2) and thus deemed it necessary to adjust for its impact on our results. Figure 2 illustrates the effects of exposure to 1 μg/m^3^ of PM₁, PM₂.₅, PM₁₀, SO₂, NO₂, and O₃, as well as 10 μg/m^3^ of CO, and 10 J/m^2^ of EDD on the odds percentage change of osteoporosis, after adjusting for individual gender, age, BMI, as well as temperature and humidity.

The results depict the impact of each pollutant on osteoporosis occurrence for both single-year exposure (lag0-lag4) and average exposure over the past 5 years (lag01-lag04). Notably, for most pollutants (PM₁, PM₂.₅, PM₁₀, CO, and ozone), the five-year average exposure demonstrates a relatively substantial and consistent risk (or protective) effect on osteoporosis. Conversely, the results for single-year exposure appear less stable. In all lag periods, neither SO₂ nor NO₂ exhibited significant associations with osteoporosis. Interestingly, for particulate matter (PM₁, PM₂.₅, and PM₁₀), the risk of osteoporosis gradually increased with increasing pollutant concentration from Lag02 to Lag04, suggesting a cumulative effect of long-term exposure. Figure 2 also indicates that long-term exposure to O₃, and EDD, as related to UV radiation, appear to be protective factors against osteoporosis. Consequently, in our subsequent multivariate analysis, we incorporate EDD as a fixed adjustment factor.

As shown in Supplementary Figure S3, the coefficients in the graph represent the effects resulting from a unit increase in pollutant concentration. Specifically, PM₁, PM₂.₅, PM₁₀, SO₂, NO₂, and O₃ units were 1 μg/m^3^, CO was 0.01 mg/m^3^, and EDD was 10 J/m^2^. Adjustments were made for gender, age, BMI, temperature, and humidity. Regarding PM₁, PM₂.₅, and PM₁₀, cumulative effects resulting from 4 or 5 years of exposure demonstrated a significant association with the occurrence of osteoporosis, consistent with the observed trend in Figure 2. Notably, neither NO₂ nor SO₂ exhibited discernible cumulative effects. CO exhibited the strongest effect with a 4-year cumulative exposure. It’s important to emphasize that O₃ demonstrates significant cumulative effects only within a 5-year accumulation period.

### 3.4 Dose–response relationships between pollutants and osteoporosis

**Figure 3:**
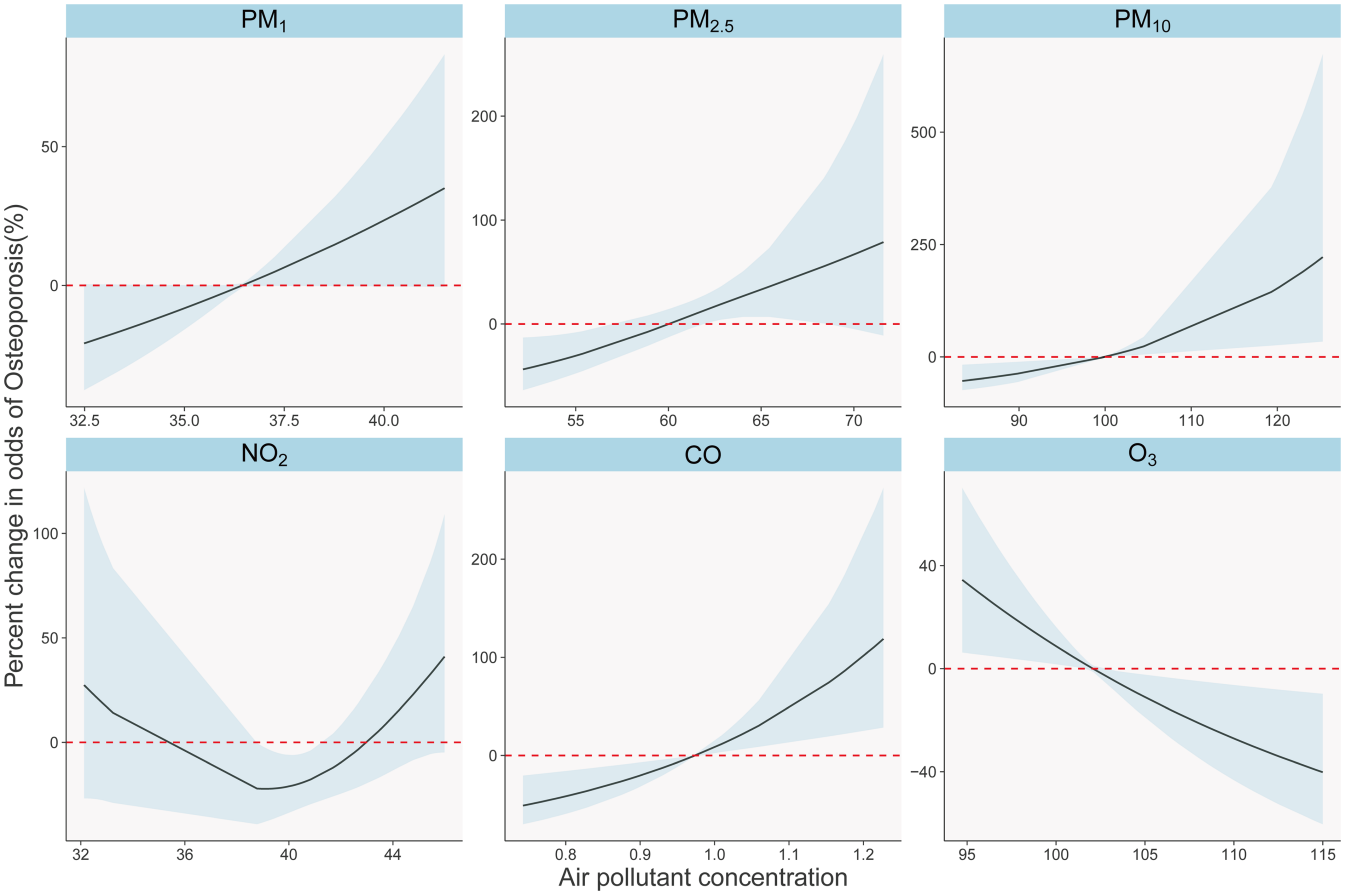
Exposure-response relationships between long-term air pollutants exposure and osteoporosis in single pollutants models. The solid black lines with shade show percent changes and 95% CI of osteoporosis odds. The dotted red lines show the referent position of 0.

In Figure 3, we present a clear depiction of the exposure-response relationship between six pollutants and the risk of osteoporosis. These relationships are adjusted for individual gender, age, BMI, as well as temperature, humidity, and EDD, considering a five-year average exposure (four-year average for PM). The concentrations of PM₁, PM₂.₅, PM₁₀, and CO exhibit a significant, nearly linear positive correlation with the risk of osteoporosis as they increase. In contrast, NO₂ demonstrates a nonlinear relationship with osteoporosis. Additionally, O₃ shows a significant negative correlation with osteoporosis occurrence.

### 3.5 Two-pollutant models

**Figure 4:**
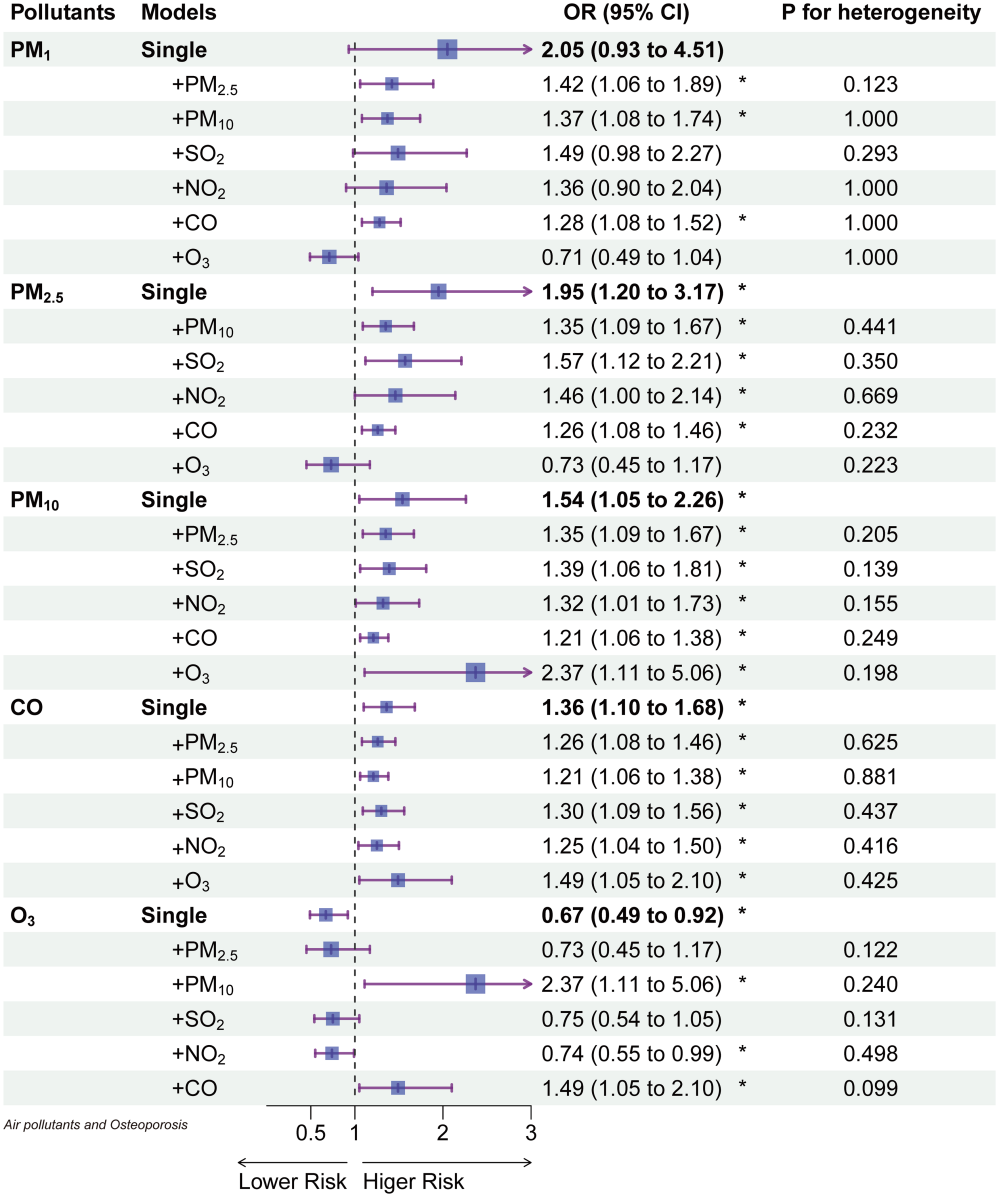
The odds ratios of osteoporosis associated with a 10 μg/m^3^ increase of each air pollutant (100 μg/m^3^ for CO) in single and 2-pollutant models.

Figure 4 presents the results of two-pollutant models for 10 μg/m^3^ of PM₁ (lag03), PM₂.₅ (lag04), PM₁₀ (lag04), and O₃ (lag04) in conjunction with 100 μg/m^3^ of CO (lag04). These models build upon the single-pollutant models by sequentially accounting for the influence of other pollutants. After adjusting for the second pollutant, the effects of PM₁₀ and CO remained relatively stable, while PM₁ and PM₂.₅ were notably influenced by O₃. However, when compared to single-pollutant models, all two-pollutant models exhibited no statistically significant differences in estimating the risk of osteoporosis occurrence (P for heterogeneity). Notably, O₃ was influenced to a greater extent by PM₁₀ and CO, with a change in effect direction after adjustment.

### 3.6 Stratified analysis

In Table 3, we present the adjusted percent change (95% CIs) for osteoporosis associated with a 1 μg/m^3^ increase in exposure to PM₁, PM₂.₅, PM₁₀, O₃, and a 10 μg/m^3^ increase in CO, stratified by age, gender, and BMI. PM₁ and PM₂.₅ showed associations with osteoporosis occurrence only among male participants (*p* < 0.05). Furthermore, their impact on osteoporosis risk in males was significantly higher than in females (*p* = 0.02). Among participants aged 60 and above, all four pollutants exhibited associations with osteoporosis, with effect sizes greater in absolute value compared to those below 60. However, these associations did not reach statistical significance. Similarly, no significant differences were observed in the associations between long-term pollutant exposure and osteoporosis across different BMI groups (*p* = 0.02). Nevertheless, it is worth noting that particle pollutants showed significant associations with osteoporosis only in individuals with a BMI greater than or equal to 25.

**Table 3:** Adjusted percent change (95% CIs) for osteoporosis associated with 1 μg/m^3^ increase of exposures to PM_1_, PM_2.5_, PM_10,_ and 10 μg/m^3^ CO stratified by age, gender, and BMI.

	Adjusted percent change (95% CIs)				O_3_
	PM_1_	PM_2.5_	PM_10_	CO	
Age					
<60	2.92 (−13.75,22.79)	0.47 (−9.88,12)	−0.37 (−7.19,6.96)	0.5 (−3.73,4.91)	−1.41 (−8.18,5.86)
≥60	10.38 (2.93,18.37)*	10.53 (4.37,17.05)*	6.84 (2.48,11.39)*	4.18 (1.61,6.82)*	−4.90 (−8.62,-1.04)*
*p* value^a^	0.47	0.13	0.1	0.16	0.39
Gender					
Male	22.24 (6.52,40.29)*	24.1 (8.98,41.31)*	7.2 (−2.02,17.3)	4.67 (−1.02,10.68)	−6.28 (−15.06,3.4)
Female	2.46 (−3.54,8.83)	4.99 (−0.83,11.15)	3.78 (−0.06,7.76)	2.64 (0.31,5.03)*	−2.75 (−6.11,0.74)
*p* value	0.02*	0.02*	0.51	0.53	0.49
BMI					
<25	5.97 (−3.98,16.96)	6.19 (−0.87,13.75)	4.06 (−0.59,8.93)	2.95 (0.12,5.85)*	−3.95 (−7.67,-0.08)*
≥25	10.97 (0.51,22.53)*	9.53 (1.61,18.07)*	6.17 (0.47,12.2)*	4.14 (0.70,7.70)*	−4.18 (−9.38,1.33)
*p* value	0.52	0.55	0.58	0.61	0.95

PM_1_, PM_2.5_, PM_10_ particulate matter with an aerodynamic diameter ≤ 1, 2.5,10 μm; CO, carbon monoxide; CI, confidence interval; BMI, body mass index. ^a^The value of *p* in a *z*-test assesses the significance of coefficient differences between two model groups. ^*^p < 0.05. The bold value were statistical significant data.

## 4 Discussion

This study presents the first evidence of a delayed effect of long-term exposure to air pollution on the occurrence of osteoporosis, with a more stable association observed at 4 to 5 years of exposure lag (lag03, lag04). The emergence of PM₁₀ as a robust indicator for assessing the relationship between particulate matter and osteoporosis is particularly noteworthy. Furthermore, our research identified individuals aged 60 and above, as well as those with a BMI of ≥ 25, as vulnerable populations to air pollutant-related osteoporosis. These significant findings offer valuable insights for further research and intervention strategies, contributing to the enhancement of public health and the formulation of environmental policies.

Our study revealed a dose–response relationship between long-term exposure to PM₁, PM₂.₅, and PM₁₀ and the risk of osteoporosis, with the odds ratios (ORs) increasing with prolonged exposure (Figure 2). More specifically, at a 5-year lag (lag04), an average increase of 1 μg/m^3^ in PM₂.₅ and PM₁₀ was associated with a 9.5 and 5.4% increased risk of osteoporosis, respectively (Figure 4). Notably, the effectiveness of PM₂.₅ was slightly higher than that found in a previous study in Hubei Province, China, which reported a 5% increased risk for every 1 μg/m^3^ increase in PM₂.₅ using a 2-year average exposure (lag01) without adjusting for temperature and humidity [OR: 1.05 (1.00, 1.11)] (21). However, they did not find a statistically significant association with osteoporosis for 1-year [OR: 1.040 (0.994, 1.088)] and 3-year [OR: 1.037 (0.990, 1.086)] average exposures, highlighting the necessity of correcting for meteorological factors and presenting lag effects comprehensively. Our results corroborated the findings of an analysis from the UK Biobank (10), which found a 9% increased risk of osteoporosis associated with a 1 interquartile range (IQR) increase (1.3 μg/m^3^) in PM₂.₅ during the follow-up period [HR: 1.09 (1.06, 1.12)]. Another report using UK Biobank data supported our results (22), showing a 94% increased risk of osteoporosis for a 10 μg/m^3^ increase in PM₁₀ [HR: 1.94 (1.52, 2.48)], with their PM₂.₅ exposure levels ranging from 8.2 to 21.3 μg/m^3^, averaging 9.9 μg/m^3^. This highlighted the linear relationship between PM₂.₅ and osteoporosis risk observed in our study (Figure 3), even at lower concentration levels. Regarding PM₁₀, the UK Biobank results demonstrated a 4% increased risk of osteoporosis associated with a 2.4 μg/m^3^ increase [HR: 1.04 (1.01, 1.07)] (10), consistent with our findings using lag0 (Figure 3). Therefore, our Figure 2 served as a valuable reference for explaining differences in similar studies. Additionally, studies from South Korea (23) and Italy (24) reported associations between PM₁₀ exposure and increased osteoporosis risk, but they employed different categorization methods for PM₁₀ and did not report specific dose–response relationships. Furthermore, the Korean study (23) did not find an association between PM₂.₅ and osteoporosis.

While research on PM₁ was relatively limited (11, 12), our study revealed that after adjusting for EDD (Erythemal Daily Dose), the impact of PM₁ on osteoporosis lacked statistical significance (Figure 4). In contrast, when not adjusting for EDD, PM₁ remained a risk factor (Figure 2), and the effect of PM₁ per unit dose was even more pronounced. Furthermore, it’s worth noting that a study employed a 3-year average PM₁ concentration and found a correlation with a −5.38 unit decrease in quantitative ultrasound index (95% CI: −6.17, −4.60) (21), this harm had already been reflected in PM₂.₅ and PM₁₀. Research on rural populations in Henan, China, also discovered that a 1 μg/m^3^ increase in the three-year average of PM₁, PM₂.₅, and PM₁₀ resulted in a 14.9, 14.6, and 7.3% higher risk of osteoporosis, respectively (25). It’s important to highlight that the efficacy of these pollutants in their study surpassed our findings, possibly due to their use of quantitative ultrasound bone density measurements to assess osteoporosis (25).

The association between PM and osteoporosis was attributed to their ability to penetrate the lower respiratory tract, exerting both direct and indirect harmful effects on various organs and tissues. These harmful effects stemmed from PM components’ capability to traverse respiratory membranes, gaining access to the bloodstream. The direct effects resulted from PM components’ ability to traverse respiratory membranes and enter the bloodstream, whereas the indirect effects encompassed systemic consequences of localized airway reactions, which involved four potential mechanisms reported in the literature: inflammation, vitamin D, oxidative damage, and some environmental endocrine disruptors (26).

In gaseous pollutants, we observed a relatively stable association between CO and osteoporosis (Figure 4). Previous research has reported a negative correlation between CO exposure and BMD T-scores in a study from Taiwan (27). Furthermore, a prior study based on healthcare data from Taiwan, China, found that an increase in CO exposure was associated with an increase in osteoporosis incidence from 13.58 per 1,000 person-years to 22.25 per 1,000 person-years (28). The binding affinity of CO to hemoglobin is much higher than that of oxygen (O₂) (29), which thus leads to hypoxia by reducing oxygen-carrying capacity and decreasing O₂ release to tissues (30). This hypoxia has been confirmed to reduce the growth of osteoblasts, resulting in bone thinning and osteoporosis (31).

Our study also unveiled a protective effect of O₃ against osteoporosis. This protective effect persisted even after adjusting for EDD (Erythemal Daily Dose), suggesting that O₃ may have independent effects apart from UV radiation (Figure 4). In line with our findings, a study by Lin et al. in 2022 in Taiwan (27) found a positive correlation between annual average O₃ exposure levels and BMD T-scores. Furthermore, literature searches have indicated an increasing clinical use of O₃ therapy for conditions such as disc herniation, jawbone necrosis, and pain management (32, 33). O₃ therapy has been demonstrated to promote complete healing of bisphosphonate-related jawbone necrosis by restoring normal function (32). Additionally, two separate studies involving rats have shown that O₃ has a positive impact on bone formation. One study involved cranial bone defects in rats (34), while another study with 48 rats demonstrated that O₃ therapy increased the number of osteoclasts and osteoblasts and stimulated bone regeneration (35). These combined findings suggest a physiological basis for the protective effect of O₃ against osteoporosis.

In our study, both SO₂ and NO₂ did not independently affect osteoporosis, which is consistent with research conducted in Hubei, China (21). Furthermore, we discovered a U-shaped relationship between NO₂ and osteoporosis, indicating a non-linear association that might have limited our ability to identify a clear link between them. Furthermore, a meta-analysis indicated that SO₂ exposure was associated with a non-significant increase in bone mineral density (BMD) (11).

Subgroup analysis indicated that individuals aged 60 and above were the most susceptible to air pollution-induced osteoporosis, potentially due to age-related immunosuppression, rendering them more vulnerable to environmental pollution. We also observed that males were more sensitive to the effects of PM₂.₅ and PM₁, which was consistent with previous reports that found that the non-standardized coefficient β (95% CI) between BMD T-score and each 1 μg/m^3^ increase in PM₂.₅ was higher in males than females [−0.005 (−0.011, 0.000) for males vs. −0.001 (−0.007, 0.005) for females] (27). Notably, individuals with a BMI ≥25 were more susceptible to the impact of air pollution, despite the protective effect of higher BMI against osteoporosis (Table 1). This susceptibility among lower-risk individuals could be explained by the fact that air pollution can trigger systemic inflammation and oxidative stress. Overweight or obese individuals often exhibited a chronic inflammatory state due to the presence of inflammatory cells and mediators in adipose tissue (36). This chronic inflammation may have heightened their sensitivity to the detrimental effects of air pollutants, as inflammation can increase cellular susceptibility to the harmful effects of gasses and particulate matter.

This study is the first to correct for the influences of both humidity and solar radiation in quantitatively assessing the correlation between air pollutants and osteoporosis. Our study also has several strengths. Firstly, we employed DXA, the gold standard for diagnosing osteoporosis, to assess bone density at six sites. This was executed meticulously through a rigorous process of stratified random sampling and the use of standardized equipment. Furthermore, we diligently standardized the equipment across all hospitals involved in the project, a crucial step that ensured the uniformity and reliability of our test results. Secondly, we also considered temperature, humidity, and ultraviolet radiation in our comprehensive analysis of air pollution and osteoporosis. Finally, for the first time, we showed how osteoporosis risk varies with different pollutants and lag times. Our study strongly indicates that as exposure duration to pollutants increases, the osteoporosis risk per unit dose of pollutants fluctuates.

Our study still has some limitations, primarily the relatively small sample size. Conducting active monitoring using DXA measurement, while ensuring result reliability, constrained our sample size. The present research cohort size has already enabled us to identify a statistically significant correlation between exposure to air pollutants and osteoporosis. While a larger sample size may bolster the observed correlation between short-term exposure and osteoporosis, it is unlikely to alter our established conclusion that the association is notably stronger with long-term exposure. However, extending the conclusion to a broader scope might necessitate a wider range of exposure to pollutants, thereby gaining further insights into the health effects at higher or lower concentrations. In the future, we plan to obtain national data from all participants in our project for further analysis. Second, due to limited air pollution data availability, we could only access data from 2013 onwards, limiting our analysis of longer exposure lags on osteoporosis. Finally, despite our best efforts to adjust for confounding factors, we cannot eliminate residual confounding, especially since factors influencing osteoporosis and bone mineral density are not yet fully understood.

Conclusively, this study reveals a potential link between air pollutants and osteoporosis, particularly emphasized with prolonged exposure. The susceptibility to air pollution-induced osteoporosis seems heightened in individuals aged 60 and above, those with a BMI exceeding 25, and among males. These findings identify specific demographics requiring targeted public health interventions to mitigate the adverse effects of air pollution on their bone health.

## Data availability statement

The original contributions presented in the study are included in the article/Supplementary material, further inquiries can be directed to the corresponding authors.

## Author contributions

HS: Conceptualization, Formal analysis, Funding acquisition, Supervision, Writing – original draft, Writing – review & editing. YW: Conceptualization, Data curation, Methodology, Writing – original draft, Writing – review & editing. XP: Investigation, Writing – review & editing, Supervision. WY: Investigation, Writing – review & editing. JS: Investigation, Resources, Writing – review & editing. JL: Investigation, Writing – review & editing. GZ: Investigation, Writing – review & editing. XL: Investigation, Writing – review & editing. XX: Investigation, Resources, Writing – review & editing. YZ: Conceptualization, Data curation, Investigation, Resources, Writing – original draft, Writing – review & editing.
