# Supplementary material for: Long-term air pollution and adverse meteorological factors might elevate the osteoporosis risk among adult Chinese
Source: Front Public Health. 2024 Jan 29;12:1361911. doi: 10.3389/fpubh.2024.1361911 (PMC10859497; doi:10.3389/fpubh.2024.1361911)
Supplement: Supplementary file 1 [file Data_Sheet_1.DOCX]

**SUPPLEMENTAL MATERIAL**

**Long-term air pollution and adverse meteorological factors might elevate the osteoporosis risk among adult Chinese**

**Hong Sun1,†, Yanan Wan1,†, Xiaoqun Pan1, Wanxi You2, Jianxin Shen3, Junhua Lu4, Gangfeng Zheng5, Xinlin Li6, Xiaoxi Xing7 and Yongqing Zhang1,***

**Figure S1 County/District Distribution Map of Survey Locations**

**
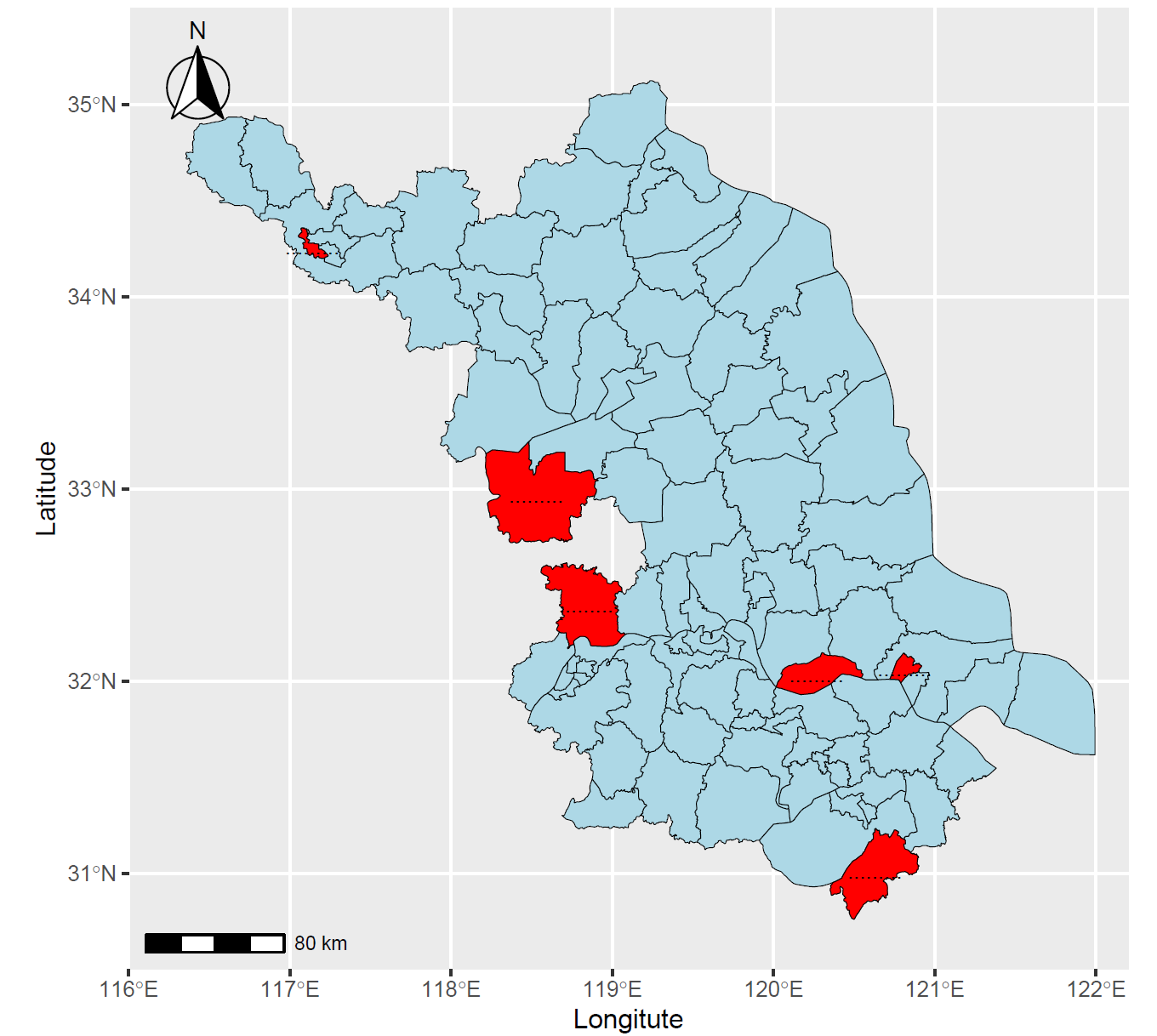
**

The areas marked in red on the map represent the 6 counties where this study was conducted.

**Figure S2 Humidity-Osteoporosis Lag & Dose-Response Charts**


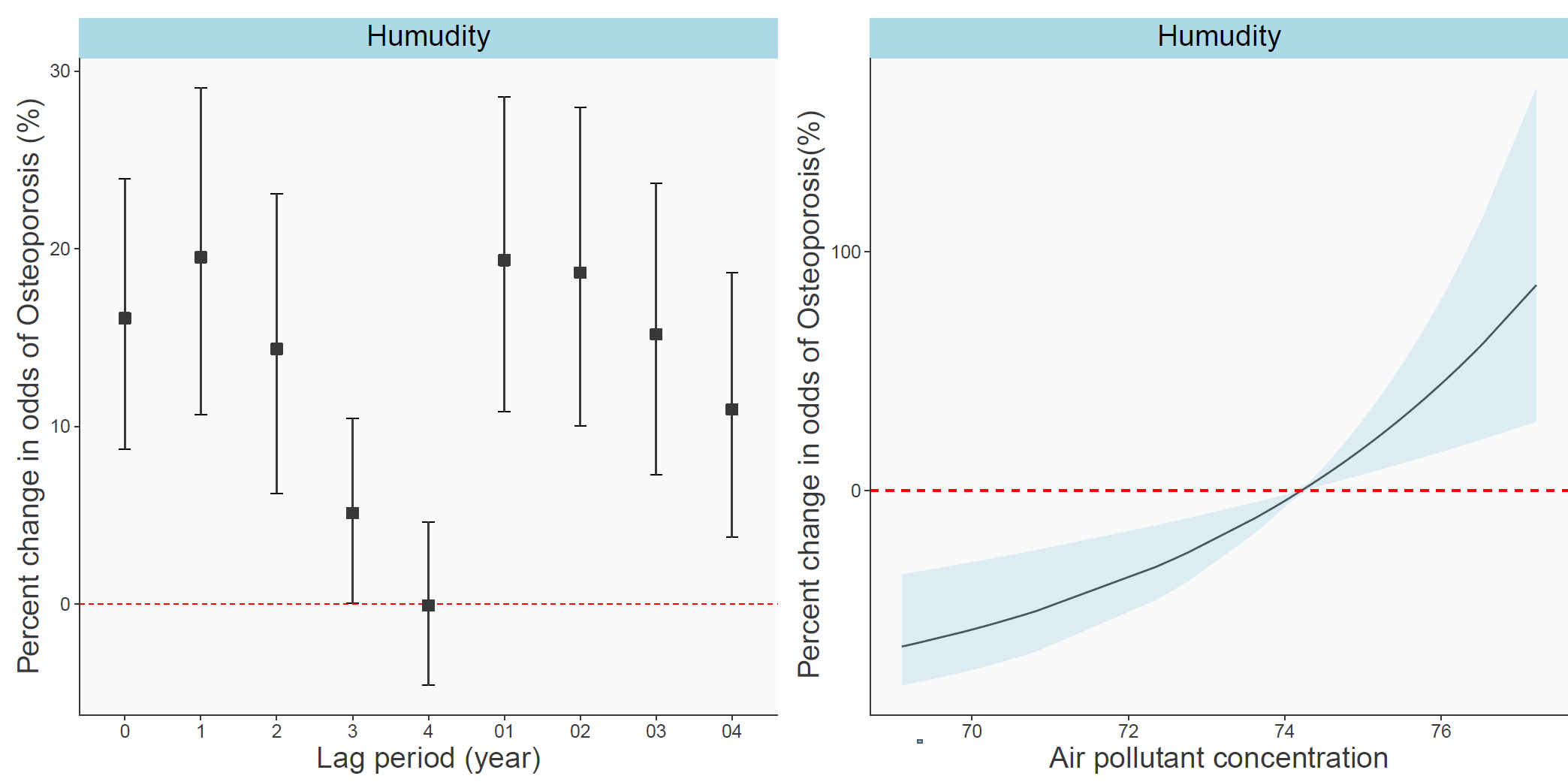


The model has adjusted for temperature, individual age, gender, and BMI.

Lag0 year: The average exposure in the year immediately before the survey day.

Lag01 (~04) year: The average exposure over the 2 years (~5 years) preceding the survey day.

**Figure S3 Cumulative Lagged Exposure Effects Over Different Years**


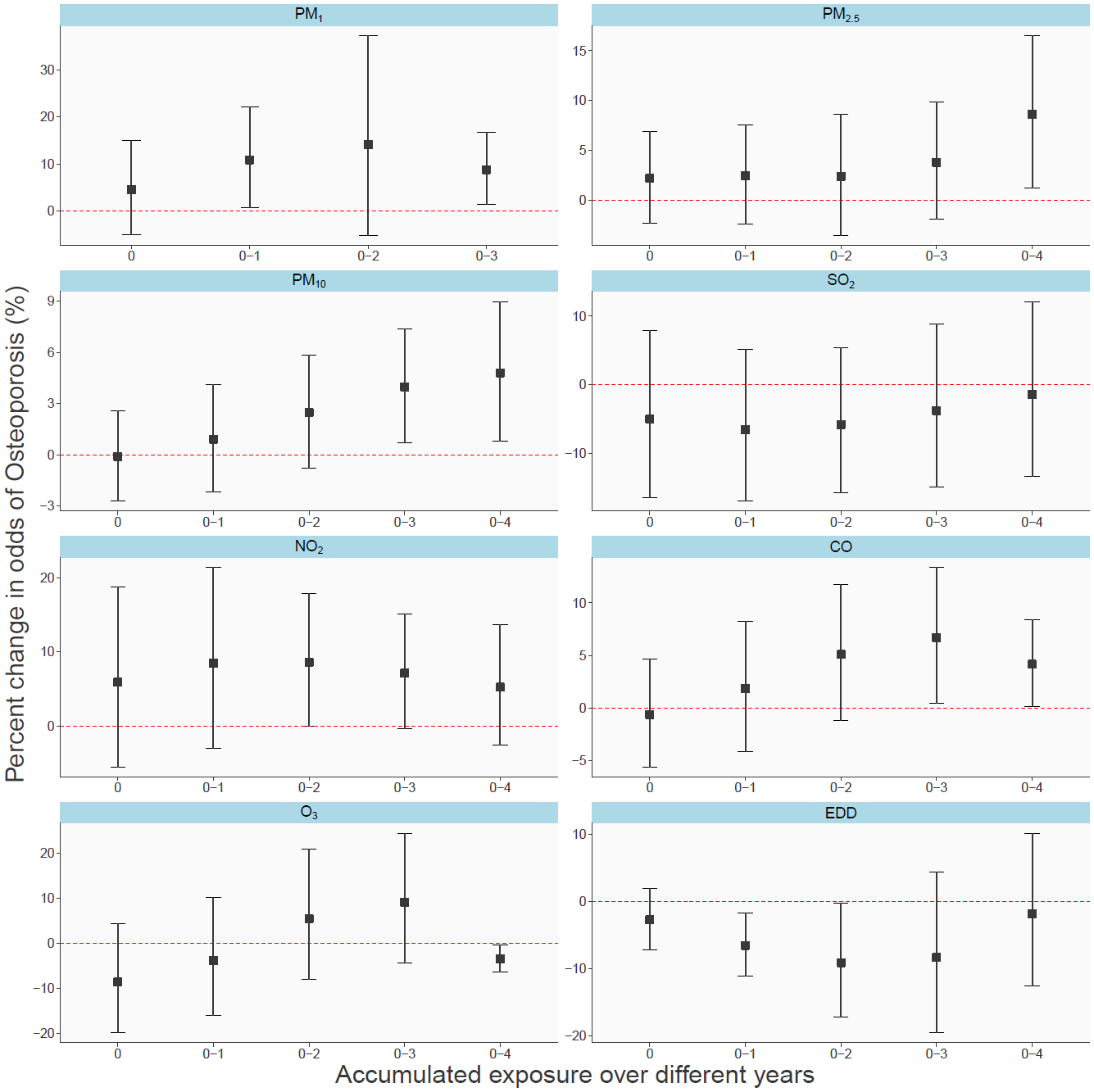


We applied the DLNM model to assess the cumulative effects of 5-year exposure to seven pollutants and EDD (Erythemal Daily Dose) on osteoporosis incidence. Coefficients in the graph represent the effects resulting from a unit increase in pollutant concentration. Specifically, PM₁, PM₂.₅, PM₁₀, SO₂, NO₂, and O₃ units were 1 μg/m³, CO was 0.01 mg/m³, and EDD was 10 J/m². Adjustments were made for gender, age, BMI, temperature, and humidity. Notably, when EDD was used as an exposure variable, it was not adjusted within the model. The x-axis represents different lag years, where 0 represents the exposure effect in the year immediately before bone density examination, and 0-n represents the cumulative effect of exposure in the previous n years on current osteoporosis. Lag0 results slightly deviate from those in Figure 2, which can be attributed to the different exposure baselines utilized.
